# Widowhood mortality among married and cohabiting partners: a nationwide study in Finland

**DOI:** 10.1093/geronb/gbaf164

**Published:** 2025-09-11

**Authors:** Marko Korhonen, Janne Lehto, Ina Rissanen

**Affiliations:** Department of Economics, Oulu Business School, University of Oulu, Oulu, Finland; Department of Economics, Oulu Business School, University of Oulu, Oulu, Finland; Research Unit of Population Health, Faculty of Medicine, University of Oulu, Oulu, Finland; Julius Center for Health Sciences and Primary Care, University Medical Center Utrecht and Utrecht University, Utrecht, The Netherlands

**Keywords:** Bereavement, Natural death, Unnatural death, Repartnering, Sex differences

## Abstract

**Objectives:**

Widowhood mortality is typically studied among those widowed from marriage, with limited knowledge about its effects after the loss of a cohabiting partner. This study examines differences in widowhood mortality by: (1) natural vs. unnatural deaths, (2) marital vs. cohabiting partnerships, (3) repartnering status, and (4) sex.

**Methods:**

Data were gathered from the registers of Statistics Finland for all adults aged 40–65 years in 1995 (*N* = 318,351), encompassing details on family relations and mortality between 1995 and 2020. Using propensity score matching, each widow(er) was matched with four control individuals based on their characteristics. Widowhood mortality was studied with Cox regression models across three time intervals: 0–3, 3–10, and 10–20 years post-widowing.

**Results:**

In the study sample, 53,852 were widowed from a marriage, 214,402 were their controls, 10,069 were widowed from a cohabiting partnership, and 40,028 were their controls. For both women and men, unnatural causes of widowhood led to a higher mortality than natural causes. The association was greater among the widowed from a cohabiting partnership than among the widowed from a marriage. Repartnering led to lower mortality than staying unpartnered after widowhood. The patterns persisted up to twenty years after partner bereavement, although the effects diminished over time.

**Discussion:**

Our findings imply that interventions for widowed individuals should be tailored depending on the cause of death (natural or unnatural) and the nature of the partnership (married or cohabitating). Future research should examine possible mechanisms to explain these effects, such as potential biological mechanisms and reduced socioeconomic security.

Numerous studies have documented that widowhood is associated with elevated mortality risks (Manzoli et al., 2007; ­Martikainen & Valkonen, 1996; Moon et al., 2011; Shor et al., 2012). There are several potential mechanisms by which widowhood contributes to mortality (Barrenetxea et al., 2023; Sullivan & Fenelon, 2014). Biologically, widowhood is known to induce stress, which is linked to cardiovascular and mental health declines (Hopf et al., 2020; Seiler et al., 2020).

From a social perspective, widowhood disrupts the primary source of emotional, instrumental, and financial support provided by the partner. Marriage, particularly in older adulthood, serves as a cornerstone of support, which directly influences health outcomes by ensuring better monitoring of health conditions, healthier behavior patterns (such as diet and exercise), and improved access to healthcare (Ding et al., 2021; Pandey et al., 2019). Moreover, the loss of partner companionship often leads to loneliness, a known risk factor for both mental and physical health problems (Yang & Gu, 2021).

Psychologically, widowhood can significantly impact an individual’s mental health (Schaan, 2013; Xu et al., 2020). The psychological trauma of losing a partner can lead to maladaptive health behaviors such as smoking, excessive alcohol ­consumption, or neglecting medical care (Zisook et al., 1990).

Furthermore, the stress-buffering model (Cohen & Wills, 1985) suggests that the availability of social support can mitigate the health impact of widowhood by buffering the stress response. Individuals with strong social networks may be able to better cope with the emotional and practical challenges of widowhood, potentially reducing the risk of depression and physical illness. However, this protective effect may vary by gender and age, as men are often more vulnerable to the loss of social support and may lack other sources of emotional or instrumental help (Christenson & Johnson, 1995). Women, by contrast, typically maintain larger social networks and may have more support from family and friends, reducing their mortality risk post-widowhood (Collins, 2018; Cudjoe et al., 2020).

Additionally, the duration of widowhood is also critical in understanding its impact on mortality. Research has suggested that the effects of widowhood on health may be stronger in the short term, particularly in the months following the death of a partner (Moon et al., 2014). However, the association between widowhood and mortality can persist over time, especially in cases where the widow or widower has limited social support or faces financial strain due to the loss (Skulason et al., 2012).

Finally, not all consequences of widowhood are negative. In some cases, the end of caregiving responsibilities, particularly for those who cared for an ailing partner, may reduce psychological and physical strain (Goda et al., 2013). Some widows and widowers may re-engage in social activities or receive increased support from friends and family, mitigating some of the adverse effects of bereavement (Costa, 1999; McGarry & Schoeni, 2005).

Widowhood mortality is typically studied solely within the context of married couples, while the number of unmarried cohabiting partners has been steadily increasing, especially in liberal Western countries (Sánchez Gassen & Perelli-Harris, 2015). Partner bereavement brings similar life changes for both married and unmarried couples, potentially resulting in comparable effects on mortality. However, unmarried individuals who experience a loss of a partner may encounter more significant difficulties due to legal and financial disadvantages. Additionally, many welfare state policies are designed around marriage and may not extend to unmarried partners (Sánchez Gassen & Perelli-Harris, 2015). This disparity can be especially pronounced in cases of an unnatural death. When death is anticipated, such as in the case of a long-term disease, couples have more time to arrange legal and financial matters in advance.

There is also a clear sex/gender difference in widowhood mortality. Research indicates that widowhood mortality is more pronounced in men than in women (Moon et al., 2011), in addition to men’s lower overall life expectancy (Knudsen et al., 2019). However, women, who often spend less time in paid work and earn less than their partners, are at a heightened risk of facing financial strains following partner bereavement (Fadlon et al., 2020). Additionally, the expectedness of partner bereavement appears to have different effects on married women and men. Men whose wives die unexpectedly exhibit higher mortality rates than those whose wives die after prolonged illness (Sullivan & Fenelon, 2014). Conversely, for women, there is no discernible difference between expected and unexpected partner bereavement.

To our knowledge, no studies have used longitudinal data to examine how natural and unnatural partner losses affect men and women in cohabiting but unmarried relationships. Overall, the existing literature on partner bereavement and mortality among cohabiting partners is limited (Blanner et al., 2020). Research indicates that one-tenth of widows and one-third of widowers form new unions within ten years after partner bereavement (Wu et al., 2015). Additionally, remarried individuals have higher mortality than first-time married individuals (Berntsen & Kravdal, 2012), showing residual widowhood mortality after remarrying. However, comparisons to cohabiting partners or unpartnered widows are lacking.

Most of the previous studies have not distinguished between married and cohabiting individuals, which leaves an important gap in understanding the differential impacts of widowhood based on relationship type. In Finland, where cohabitation is common, with cohabiting couples representing about 20% of all partnered families (Official Statistics of Finland, 2023), and often a long-term living arrangement, the health trajectories of widowhood may differ substantially for cohabiting individuals. In this study, we define a cohabiting couple as two unmarried adults of different sexes aged 18 and over who occupy the same dwelling on a permanent basis, provided their age difference is less than 16 years, and they are not siblings. Cohabitation has become increasingly common in Finland over the past few decades. However, older individuals, especially those with higher education, are more likely to marry when they form a partnership. At the same time, there is a growing trend of second marriages and long-term cohabitation among older people in Finland as well. The relationship between cohabitation and marriage formation is less strong in Finland than in other countries, and many couples remain in a long-term cohabiting relationship without marriage.

A major concern in studying the impact of widowhood on mortality is selection bias. Marriage selection bias arises because individuals who marry or remain unpartnered differ systematically in ways that may influence health and longevity (Li et al., 2023; Lillard & Panis, 1996). Married individuals tend to share similar characteristics, such as age, education, and health behaviors. If healthier or more socioeconomically advantaged individuals are more likely to get married and stay married, then the observed lower mortality rates among married individuals could be due to this pre-existing selection rather than a causal effect of marriage itself (Espinosa & Evans, 2008; Requena & Reher, 2021). For example, if individuals who maintain healthier lifestyles are more likely to marry, then the observed mortality difference between married and unpartnered individuals might reflect this selection rather than an actual protective effect of marriage. This suggests that health-­related lifestyle choices are not randomly distributed among partnered individuals.

Further, empirical estimates of the widowhood effect may be affected by selection bias, to the extent that individuals who are more likely to become widowed, such as those in older age or with pre-existing health conditions, also face a different risk of mortality independently of bereavement. This may partially confound estimates of the causal effect of widowhood on mortality. In addition, because individuals tend to partner with others of similar socioeconomic status (i.e., assortative mating), those with lower socioeconomic status are often doubly exposed, both to higher mortality risk themselves and to an increased risk of experiencing the death of a partner (Elo, 2009; Tarkiainen et al., 2012). We should note that some previous studies suggest widowhood mortality is not solely explained by selection bias, but that the death of a partner has a causal effect on mortality (Elwert & Christakis, 2008b; Martikainen & Valkonen, 1996).

In this study, we examine widowhood mortality across sexes, considering both natural and unnatural deaths, as well as repartnering, in the context of married vs. cohabiting partners, using a nationwide sample from Finland. To mitigate selection bias, we employ propensity score matching to account for the influences of age, socioeconomic status, children, and living conditions 2 years before partner bereavement. Our analysis spans widowhood mortality from 7 days to 20 years after partner bereavement.

## Method

### Study population

Data from all Finnish adults who lived in Finland, were married or cohabiting with the opposite sex, and were aged between 40 and 65 in 1995 were collected from the national registers of Statistics Finland (*N *= 318,351). The data encompassed information on income, residency, education, family relations, and mortality spanning the years 1995–2020 (Official Statistics of Finland, 2024a, 2024b, 2024c, 2024d). The study population was followed from the moment of partner bereavement, at the earliest on January 1, 1995, until their death, or until December 31, 2020, whichever came first.

### Relationship data

Married couples were identified from family relation data of Statistics Finland. The data included complete information on wedding dates and divorce dates. Our focus was on the first observed marriages within our dataset, directly identified from data, although not required that the marriage was their first relationship. Married couples were not required to live at the same address or to stay married until the end of the follow-up. Same-sex marriage was only legalized in Finland in 2017, and therefore, only opposite-sex marriages were considered.

We defined a cohabiting couple as two unmarried adults of different sexes aged ≥18 and living in the same address, provided their age difference was less than 16 years, and they were not siblings. The Statistics Finland data included the commencement and conclusion dates of cohabitation from 1987 onward. The first cohabitations were identified as the initial observed cohabitations.

### Cause of widowhood

Mortality data encompassed information on death dates and causes of death. Using the death dates, we identified individuals who became widows or widowers during the study period. Specifically, widows and widowers were those who were married to their spouse or cohabiting partners, and for whom we did not observe a divorce or death before the death date of their partner. Deaths were categorized as natural or unnatural following the classification of Statistics Finland (Official Statistics of Finland, 2024a), based on International Classification of Diseases version 10 codes for causes of death. Natural deaths included those caused by malignancies, cardiovascular diseases, pulmonary diseases, metabolic diseases, dementia, other neurological diseases, digestive diseases excluding alcohol-related causes, urinary diseases, and infections. Unnatural deaths encompassed those caused by accidents or violence, suicide, and alcohol-related causes.

### Propensity score matching

Propensity score matching was employed to match each individual widowed from a marriage and an individual widowed from a cohabiting partnership with four individuals as controls based on individual characteristics two years prior to widowhood. The matching criteria were exact marital status (married or cohabiting partner), precise age in the matching year (same year of birth), exact sex, educational level, number of children under 18 living in the household, income percentile, and the degree of urbanicity of the living environment. Educational level was categorized into two classes: having a university or applied university degree vs. not. The income percentile was used as a continuous variable. Urban-rural classification was used in two classes: living in a city center vs. living in any other area. All information used in matching was obtained from Statistics Finland. No imputation of missing values was performed for any of these variables. Due to the stringent requirements for exact matching on marital status, age, and sex, it was not feasible to match some widows with four controls. These widows were matched with fewer control individuals and retained in the analysis. Our sample includes 1,006 fewer controls for married couples (0.47% of controls), and 248 fewer controls for cohabiting partners (0.62% of controls) than in a complete 1:4 matching scenario, where each widow would have four matched controls. We used the PROC PSMATCH Procedure in SAS for matching.

### Outcome

The outcome was all-cause mortality. Mortality data from Statistics Finland, including information on death dates, were used as the outcome measure (*Official Statistics of Finland: Deaths*).

### Statistical analyses

Associations between widowhood, natural and unnatural causes of widowhood, repartnering, and mortality were analyzed with Cox proportional hazards models. The main models were done separately for married and cohabiting individuals. As sensitivity analyses, the differences between marital classes regarding natural and unnatural causes of widowhood were analyzed, combining the married and cohabiting partners into the same model. All analyses were conducted separately for women and men. Results are presented as hazard ratios (HR) and their 95% confidence intervals (CIs). Time-in-study was used as the time scale in the analyses. Age and age squared were included as covariates in all Cox regressions to control for increased mortality risk through age. Widowhood mortality was investigated within three distinct time periods: 7 days to 3 years, 3–10 years, and 10–20 years after widowing. This division was implemented due to the violation of the proportionality hazard assumption in Cox regression (Figure S1A-J, see online supplementary material).

## Results

### Characteristics of the sample

The study sample consisted of 318,351 individuals, including 53,852 widowed from a marriage and their 214,402 controls; and 10,069 widowed from a cohabiting partnership and their 40,028 controls. The total follow-up was 3,567,075 person years.

Married individuals were older than cohabiting partners, had better incomes, and were more likely to be living in rural environments (Table 1). Women comprised 73% of married and 68% of cohabiting partners. Accidents, violent deaths, and alcohol-related deaths were more prevalent causes of widowhood among cohabiting partners than among married ones. In contrast, married individuals were more likely to have been widowed due to cancer (Table S1, see online supplementary material).

**Table 1.** Baseline characteristics of the sample.

Characteristics	Widowed from a marriage (*n *= 53,852)		Married individuals (*n *= 214,402)		Widowed from a cohabiting partnership (*n *= 10,069)		Cohabiting partners (*n *= 40,028)	
	Mean (*SD*)	%	Mean (*SD*)	%	Mean (*SD*)	%	Mean (*SD*)	%
**Women**		72.6		72.6		67.6		67.6
**Age**	54.5 (6.5)		54.5 (6.5)		52.6 (6.9)		52.6 (6.9)	
**Person years of follow-up**	11.1 (6.6)		11.50 (6.6)		9.65 (6.3)		10.3 (6.5)	
**Number of children <18 years living in the same household**	0.6 (0.9)		0.7 (1.0)		0.4 (0.8)		0.7 (0.9)	
**Income percentile**	51.5 (29.0)		47.2 (32.9)		43.1 (28.3)		43.8 (33.2)	
**Deceased partner’s income percentile**	47.8 (29.1)		21.3 (37.5)		36.7 (27.5)		29.5 (42.7)	
**University degree**		17.8		20.0		11.2		32.4
**Urban living**		26.1		20.0		30.9		32.4
**Cause of widowhood**								
** Natural**		81.3		n.a.		71.6		n.a.
** Unnatural**		17.6		n.a.		27.3		n.a.
**Repartnering after widowhood**								
** New marriage**		7.4		n.a.		6.7		n.a.
** New cohabiting**		10.8		n.a.		12.9		n.a.
**Died during follow-up**		11.4		7.8		14.5		7.1
**Mortality rate per 100,000 person years**	1,025		679		1,506		692	
**Cause of own death**								
** Natural**		9.3		6.8		10.2		5.6
** Unnatural**		1.9		0.8		4.1		1.5

### Widowhood mortality

Over the follow-up period, 27,132 persons died. Mortality rates as per 100,000 person years were 1,025 for widowed from a marriage, 679 for their controls, 1,506 for widowed from a cohabiting partnership, and 692 for their controls (Table 1).

**Figure 1.**
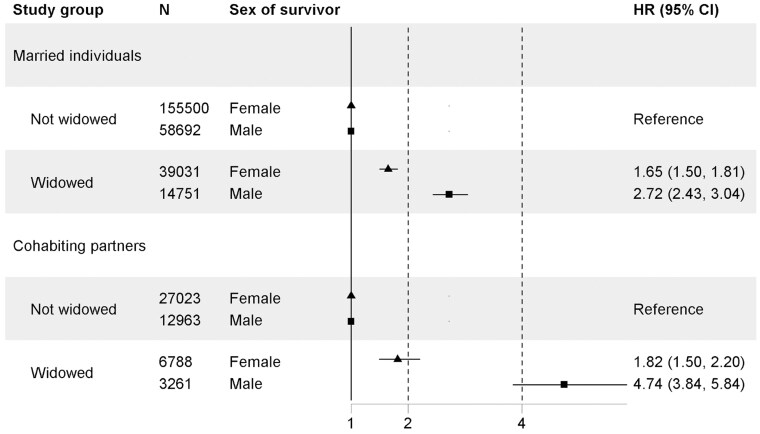
Widowhood mortality during the first 3 years after widowhood among married individuals and cohabitant partners, stratified by sex. CI = confidence interval; HR = hazard ratio.

Widowhood increased one’s mortality 1.65-fold (95% CI 1.50-1.81) among married women and 2.72-fold (95% CI 2.43-3.04) among married men during the first three years of widowhood, compared to their controls (Figure 1). Accordingly, widowhood increased mortality 1.82-fold (95% CI 1.50-2.20) among cohabiting women and 4.74-fold (95% CI 3.84-5.84) among cohabiting men compared to their controls. The same pattern was found up to 20 years of widowhood (Figure S2 and S3, see online supplementary material). Widowhood mortality related to specific causes of widowhood is shown in Figure S4 (see online supplementary material).

**Figure 2.**
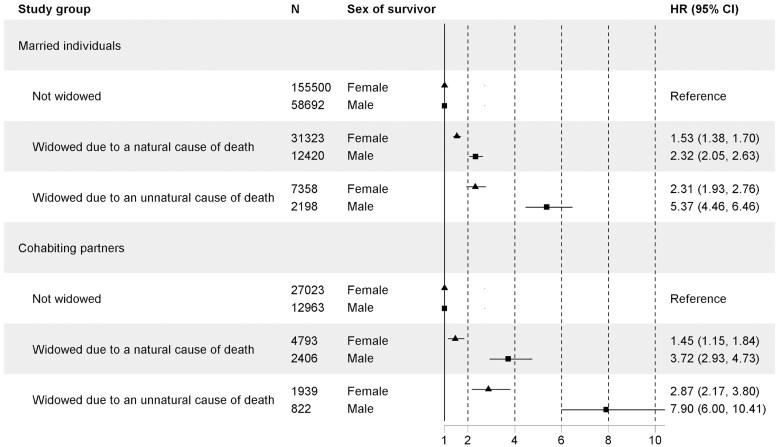
Widowhood mortality during the first 3 years after widowhood among married individuals and cohabitant partners, stratified by sex and cause of widowhood. CI = confidence interval; HR = hazard ratio.

In all studied groups, the unnatural cause of widowhood was associated with greater mortality than the natural cause of widowhood (Figure 2). However, the effect size was larger among men. During the first 3 years of widowhood, married men with unnatural causes of widowhood faced a 5.4-fold (95% CI 4.5–6.5) mortality hazard compared to controls. For cohabiting men, the hazard was 7.9-fold (95% CI 6.0–10.5). The same pattern was found up to 20 years after widowhood; however, the hazards were lower (Figures S5 and S6, see online supplementary material).

**Figure 3.**
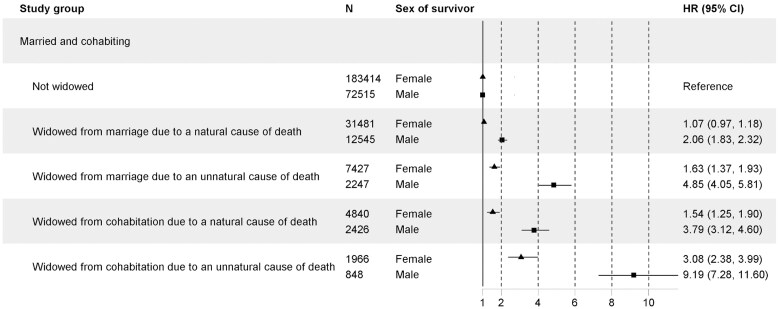
Widowhood mortality during the first 3 years after widowhood comparing widowed from a marriage and widowed from a cohabiting partnership to all controls, stratified by sex and cause of widowhood. CI = confidence interval; HR = hazard ratio.

In the additional analyses where all the controls were pooled together as the reference group, the highest mortality hazard was found in cohabiting men whose widowhood was of an unnatural cause (HR 9.19; 95% CI 7.28–11.60) (Figure 3). Similarly, among women, cohabiting partners whose widowhood was of an unnatural cause had also the highest mortality compared to controls (HR 3.08; 95% CI 2.38–3.99). Again, the same pattern was found up to 20 years after widowhood with lower hazards (Figure S7 and S8, see online supplementary material).

### Repartnering

**Figure 4.**
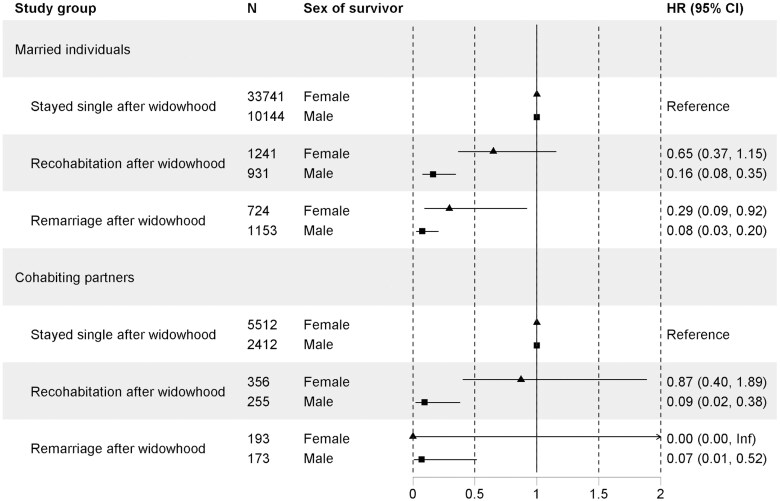
Widowhood mortality during the first 3 years after widowhood among married individuals and cohabitant partners, stratified by sex and repartnering status after widowhood. CI = confidence interval; HR = hazard ratio.

We found that 7.4% of widowed individuals from a marriage remarried after widowhood, 10.8% established a cohabiting relationship, and 81.8% stayed unpartnered. Of the widowed from a cohabiting partnership, 6.7% married after widowhood, 12.9% established another cohabiting relationship, and 80.4% stayed unpartnered. Figure 4 shows mortality hazards during the first 3 years after widowhood among unpartnered, re-cohabiting, and remarrying women and men. In all studied groups, repartnering, especially remarrying, was associated with decreased mortality compared with staying unpartnered after widowhood. The findings were similar up to 20 years after widowhood (Figure S9 and S10, see online supplementary material).

## Discussion

In this nationwide study of individuals aged 40–65 in Finland, we examined widowhood mortality by comparing married and cohabiting partners, natural and unnatural causes of widowhood, repartnered and unpartnered widow(er)s, and sex differences. We found that mortality following partner loss was highest when the cause of death was unnatural, for both women and men. The association was stronger for those bereaved from cohabiting relationships than from marriages. Widowhood mortality persisted for up to 20 years but declined over time. Repartnering was associated with lower mortality compared to remaining unpartnered, suggesting that the mortality effect of widowhood may be partially reversible. All findings were adjusted for age, number of children, socioeconomic status, and living conditions.

While the purpose of our study was not to identify specific reasons for differences in mortality risk between those who were married and those who were in cohabiting relationships before losing a partner, several potential mechanisms may explain these differences. First, marriage is associated with a higher socioeconomic status, especially in older age groups. Higher socioeconomic status is linked to better access to healthcare, healthier lifestyles, and stronger social support, all of which contribute to lower mortality risks after widowhood (Franke & Kulu, 2018; Lindström & Rosvall, 2019). Additionally, due to lower socioeconomic status, cohabiting individuals may have fewer financial resources or less access to spousal benefits, such as pensions or healthcare, which could contribute to higher mortality risks (Balter et al., 2023).

Second, legal protections and benefits, such as survivor pensions and inheritance rights, are stronger for married individuals than for cohabiting partners in many countries (Ehlert, 2021). This implies that cohabitants may not qualify for the same level of post-bereavement financial support, increasing economic insecurity and potentially worsening health outcomes. In Finland, cohabiting partners have a significantly weaker position than married partners. Unlike married couples, they do not have matrimonial rights to each other’s properties, inherit each other’s assets without a will, or have a right to stay in their shared home owned by the deceased. The legal regulations of cohabitation may be different in the case of death from one country to another (Sánchez Gassen & Perelli-Harris, 2015). However, in most countries, cohabiting partners face more socioeconomic insecurity than married individuals in the case of partner bereavement.

Third, marriage is often associated with stronger social networks that extend beyond the couple, including in-laws and mutual friends, which may help buffer against the negative health effects of widowhood (Anusic & Lucas, 2014). Fourth, the psychological impact of losing a partner may be different depending on the nature of the relationship. Marriage is often associated with a stronger sense of long-term commitment and shared life planning, which could lead to greater emotional distress upon widowhood but also stronger long-term coping mechanisms. Cohabitants may have been more accustomed to independent decision-making, potentially reducing the immediate psychological shock but increasing the risk of social isolation in the long run (Brown & Wright, 2017).

Additionally, selection effects may contribute to these differences. Those who choose marriage vs. cohabitation may already have different characteristics that influence post-widowhood mortality (Kulu et al., 2024). Marriage may attract individuals who are more family-oriented, financially stable, and risk-averse, while cohabitation may be more common among those with less stable relationship histories or weaker social ties (Lundberg et al., 2016) that are associated with higher mortality. While selection effects are a potential concern, prior research has shown that widowhood mortality persists even after accounting for shared partner characteristics (Boyle et al., 2011; Espinosa & Evans, 2008). For instance, one study found that the death of an ex-wife had no effect on her ex-husband’s mortality, supporting a causal interpretation of widowhood mortality (Elwert & Christakis, 2008b). Similarly, in our study, widowhood mortality remained even after adjusting for individual characteristics and cause of partner death. However, we acknowledge the possibility of residual confounding, particularly as we lacked data on lifestyle factors. Notably, unnatural and alcohol-related deaths—potential indicators of riskier lifestyles—were more common among cohabiting than married individuals.

The existing literature offers various explanations for widowhood mortality, including psychological distress, disruption of social networks, and financial strain. Some studies suggest that the psychological impact of partner loss is similar regardless of the cause of death (Carr et al., 2001), while others report no mortality increase when death was expected, such as among caregivers (Ryan, 1992). As we lacked clinical data to assess expectedness, we used cause of death (natural vs. unnatural) as a proxy. We acknowledge this is an imperfect measure, as some natural deaths can still be sudden. Nonetheless, our findings are consistent with previous research showing that widowhood mortality varies by cause of partner death (Elwert & Christakis, 2008a). Unnatural deaths are often sudden and traumatic, which may lead to more intense shock and psychological distress in the surviving partner, potentially increasing their risk of mortality. However, unnatural deaths may reflect shared risk environments or behaviors (e.g., substance use, risk-taking), which could also affect the surviving partner’s mortality.

Repartnering emerged as a protective factor. Our findings confirm previous literature showing that repartnering after widowhood is more common among men, and most repartnering occurs within ten years of widowhood or not at all (Wu et al., 2015). We found that repartnering after widowhood was associated with lower mortality among both sexes than staying unpartnered, consistent with the idea that the negative health effects of widowhood may be at least partially reversible. Of course, the finding may also reflect selection out of widowhood, in that the healthiest individuals remarry and leave the widowed state, leaving only the frailest as widow(er)s (Sullivan & Fenelon, 2014).

### Strengths and limitations

The strengths of our study include a large, comprehensive, nationwide register of data. There was no loss to follow-up. We used propensity score matching to adjust for baseline age, number of children, socioeconomic status, and living conditions. Propensity score matching, with similar findings of widowhood mortality’s independence from baseline characteristics, has been utilized in previous studies as well (Blanner et al., 2020). We accounted for family composition by matching individuals based on the number of children under 18 living at the same address. Therefore, we believe that the findings are independent of the social and financial effects related to having children. Further, we studied widowhood mortality up to 20 years following partner bereavement. Our findings are in line with previous studies showing that widowhood mortality is stronger shortly after partner bereavement (Martikainen & Valkonen, 1996; Moon et al., 2011, 2014; Shor et al., 2012), but remains raised for at least for a decade (Blanner et al., 2020; Boyle et al., 2011).

Noteworthy, we limited our analyses to individuals aged 40–65 years at baseline. Prior research indicates that widowhood mortality is significantly higher for younger widows than for older widows, implying that partner deaths may be less shocking at older ages when they become more commonplace (Blanner et al., 2020; Moon et al., 2011). Further, we exclusively focused on opposite-sex marriages and cohabiting partners, necessitating further studies to explore widowhood mortality in same-sex marriages. Third, as we identified cohabiting partners as ­opposite-sex individuals living in the same address, our data may include individuals who are cohabiting but are not necessarily partners. Fourth, we did not consider differences in widowhood mortality between ethnicities. There is evidence suggesting that widowhood mortality is stronger for non-White people (Liu et al., 2020). Fifth, our measure of the “expectedness” of deaths may mask extensive variation, particularly among natural deaths. Sixth, we did not consider the living arrangements of married individuals. Since we observed marriages based on civil status and not based on addresses, the married couples did not necessarily live together. Including married couples who were not living together may have introduced heterogeneity in the exposure to partner loss. Finally, we did not have information on Finnish citizens living abroad. While this data could have provided further insights, we believe its absence is unlikely to significantly affect the overall conclusions of the study.

In conclusion, we observed a similar pattern among women and men, with widowhood mortality being stronger in cases of unnatural partner bereavement and when the couple was unmarried. Repartnering after widowhood associated with lower mortality than staying unpartnered, suggesting a partially reversible widowhood mortality. Apart from biological mechanisms, widowhood mortality might be associated with compromised economic security. The findings suggest that socioeconomic status may act as a protective factor against mortality following widowhood. Individuals with higher socioeconomic status typically have greater access to health care services, more stable housing, and the financial means to afford private support services, such as home assistance and therapy (Sullivan & Fenelon, 2014). Higher socioeconomic status is also linked to stronger social networks, which can provide emotional support and foster social engagement, both of which are important buffers against the adverse effects of widowhood (Freak-Poli et al., 2022).

In light of these protective factors, policymakers could help reduce socioeconomic status-based disparities in widowhood outcomes by improving access to primary care, mental health services, and community-based support in lower-income neighborhoods. Strengthening pension systems and survivor benefits could help alleviate the material stressors that exacerbate health risks associated with bereavement.

## Supplementary Material

gbaf164_Supplementary_Data

## Data Availability

The data that support the findings of this study are available from Statistic Finland. Restrictions apply to the availability of these data, which were used under license for this study. The study was not preregistered. For information on accessing the data, see www.stat.fi. The authors do not have permission to share data.
