# Supplementary material for: Widowhood mortality among married and cohabiting partners: a nationwide study in Finland
Source: J Gerontol B Psychol Sci Soc Sci. 2025 Sep 11;80(12):gbaf164. doi: 10.1093/geronb/gbaf164 (PMC12597676; doi:10.1093/geronb/gbaf164)

***The Journals of Gerontology, Series B: Psychological Sciences and Social Sciences* Supplementary Material: Korhonen, Leho, & Rissanen. Widowhood mortality among married and cohabiting partners: A nationwide study in Finland.**

**Supplementary Table 1.** Causes of widowhood among married widow(er)s and widowed cohabiting partners.

| **Cause of widowhood** | **Widowed from a marriage (*n* = 53,852)** | **Widowed from a cohabiting partnership (*n* = 10,069)** |
| --- | --- | --- |
|  | **N (%)** | **N (%)** |
| **Natural** | 43,790 (81.3) | 7,211 (71.6) |
| Malignancies | 23,940 (44.5%) | 3,379 (33.6%) |
| Cardiovascular disease | 14,473 (26.9%) | 2,853 (28.3%) |
| Dementia | 1,881 (3.5%) | 237 (2.4%) |
| Digestive disease | 867 (1.6%) | 196 (1.9%) |
| Metabolic disease | 703 (1.3%) | 149 (1.5%) |
| Pulmonary disease | 976 (1.8%) | 196 (1.9%) |
| Urinary disease | 118 (0.2%) | 20 (0.2%) |
| Other diseases | 832 (1.5%) | 181 (1.8%) |
| **Unnatural** | 9,495 (17.6%) | 2,753 (27.3%) |
| Accidents and violent deaths | 3,316 (6.2%) | 897 (8.9%) |
| Alcohol-related causes | 4,010 (7.4%) | 1,333 (13.2%) |
| Suicide | 2,169 (4.0%) | 523 (5.2%) |
| **Missing, other or non-classified** | 567 (1.1% | 105 (1.0%) |

**Supplementary Table 2**. Causes of death among all individuals.

| **Cause of own death** | **Widowed from a marriage**  **(*n* = 53,852)** | **Married individuals**  **(*n* = 214,402)** | **Widowed from a cohabiting Partnership**  **(*n* = 10,069)** | **Cohabiting partners**  **(*n* = 40,028)** |
| --- | --- | --- | --- | --- |
|  | **N (%)** | **N (%)** | **N (%)** | **N (%)** |
| **Natural** | 4,993 (9.3%) | 14,652 (6.8%) | 1,027 (10.2%) | 2,233 (5.6%) |
| Malignancies | 2,156 (35.1%) | 7,283 (43.7%) | 433 (29.6%) | 1,079 (37.8%) |
| Cardiovascular disease | 1,778 (28.9%) | 4,016 (24.1%) | 389 (26.6%) | 656 (23.0%) |
| Dementia | 548 (8.9%) | 1,981 (11.9) | 61 (4.2%) | 272 (9.5%) |
| Digestive disease | 152 (2.5%) | 461 (2.8%) | 40 (2.7%) | 88 (3.1%) |
| Metabolic disease | 80 (1.3%) | 202 (1.2%) | 26 (1.8%) | 29 (1.0%) |
| Pulmonary disease | 175 (2.8%) | 326 (2.0%) | 46 (3.1%) | 48 (1.7%) |
| Urinary disease | 21 (0.3%) | 75 (0.5%) | 6 (0.4%) | 10 (0.4%) |
| Other diseases | 83 (1.4%) | 308 (1.8%) | 26 (1.8%) | 51 (1.8%) |
| **Unnatural** | 1,044 (1.9%) | 1,666 (0.8%) | 412 (4.1%) | 591 (1.5%) |
| Accidents and violent deaths | 373 (6.1%) | 653 (3.9%) | 105 (7.2%) | 171 (6.0%) |
| Alcohol-related causes | 543 (8.8%) | 787 (4.7%) | 270 (18.5%) | 348 (12.2%) |
| Suicide | 128 (2.1%) | 226 (1.4%) | 37 (2.5%) | 72 (2.5%) |
| **Missing, other or non-classified** | 197 (0.4%) | 350 (0.2%) | 24 (0.2%) | 33 (0.1%) |

**Supplementary Table 3.** Re-partnering after widowhood.

|  | | **Married** | | **Cohabiting** | |
| --- | --- | --- | --- | --- | --- |
|  |  | **Women**  **(*n* = 39,000)** | **Men**  **(*n* = 14,754)** | **Women**  **(*n* = 6,662)** | **Men**  **(*n* = 3,229)** |
|  |  | **N (%)** | **N (%)** | **N (%)** | **N (%)** |
| Single | 33,799 (86.7%) | 10,169 (68.9%) | 5,532 (83.0%) | 2,416 (74.8%) |  |
| Remarrying |  |  |  |  |  |
| 0-3 years | 724 (1.9%) | 1,153 (7.8%) | 193 (2.9%) | 173 (5.4%) |  |
| 3-10 years | 808 (2.1%) | 909 (6.2%) | 126 (1.9%) | 111 (3.4%) |  |
| 10-20 years | 143 (0.4%) | 128 (0.9%) | 19 (0.3%) | 16 (0.5%) |  |
| Recohabiting |  |  |  |  |  |
| 0-3 years | 1,241 (3.2%) | 931 (6.3%) | 356 (5.3%) | 255 (7.9%) |  |
| 3-10 years | 1,774 (4.5%) | 1,128 (7.6%) | 349 (5.2%) | 213 (6.6%) |  |
| 10-20 years | 446 (1.1%) | 246 (1.7%) | 76 (1.1%) | 27 (1.7%) |  |
| Missing | 65 (0.2%) | 90 (0.6%) | 11 (0.2%) | 18 (0.6%) |  |

**Supplementary Figure 1a-j.** Log hazard functions to show the time varying nature of widowhood mortality.

1. Log hazard ratio from the Cox model, which is estimated for females for first three years of follow-up


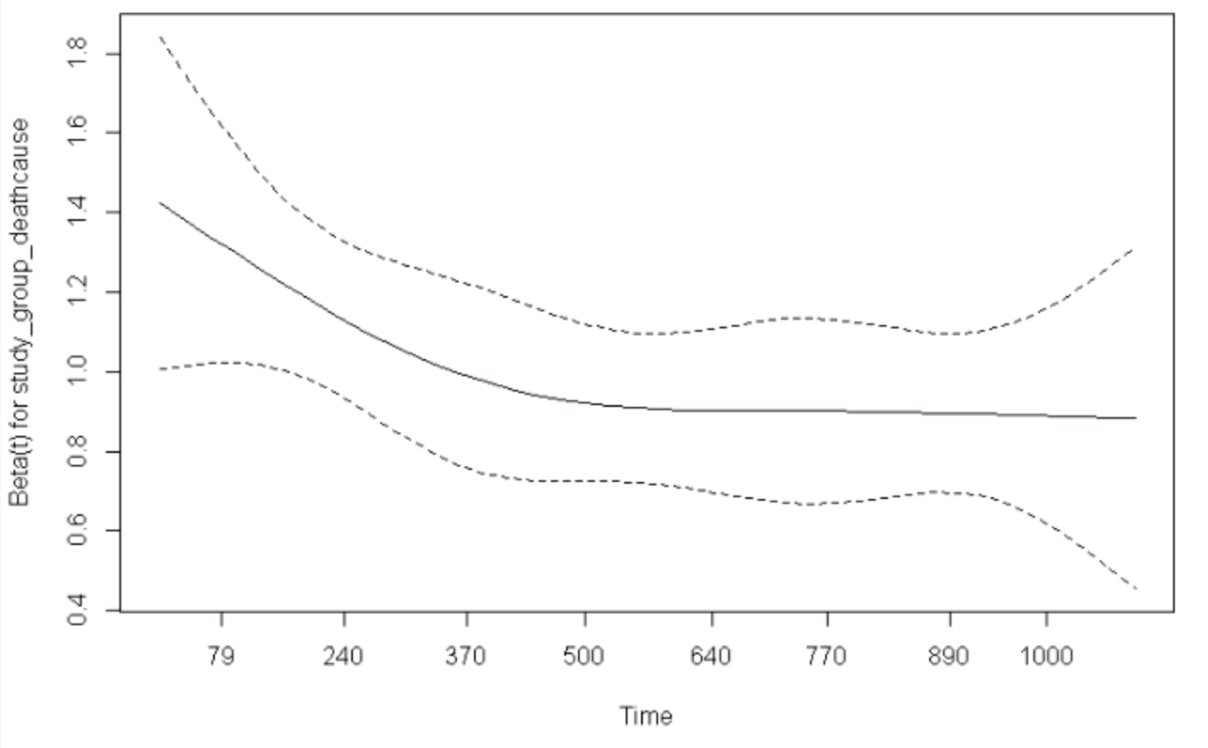


1. Log hazard ratio from the Cox model, which is estimated for males for first three years of follow-up


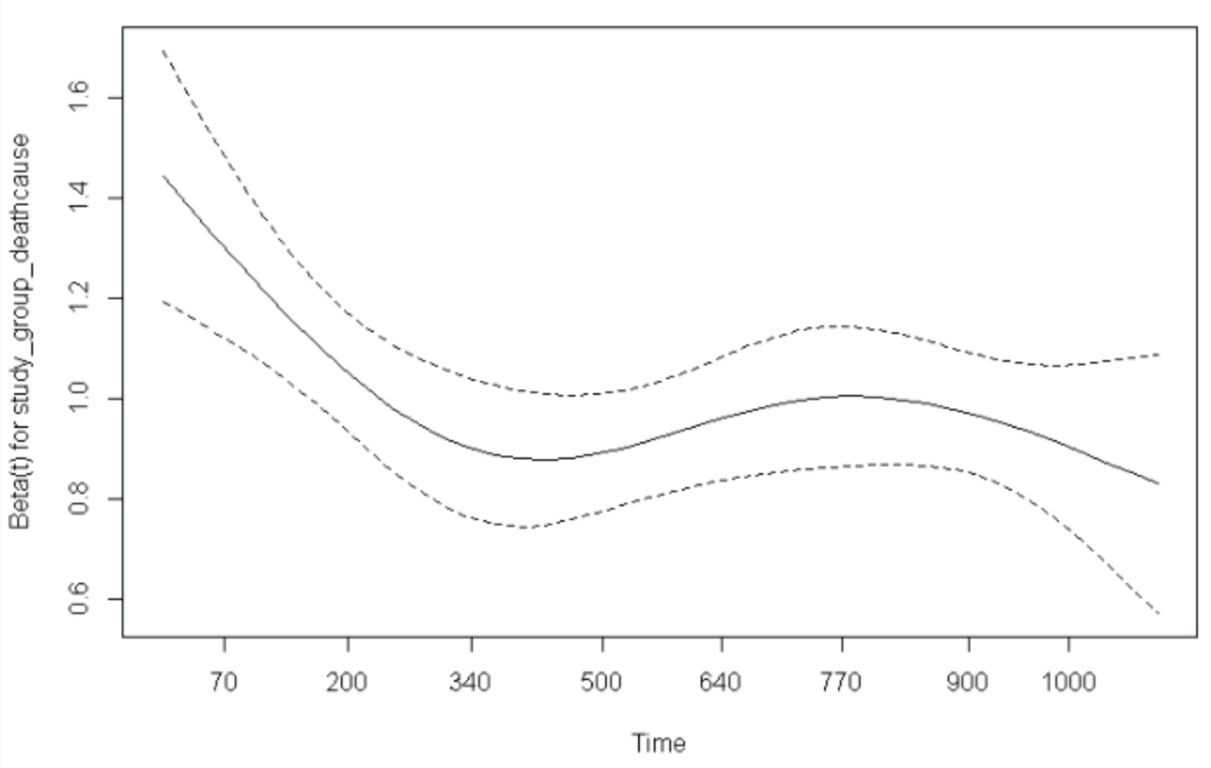


1. Log hazard ratio from the Cox model, which is estimated for females for years 3 to 10 of follow-up


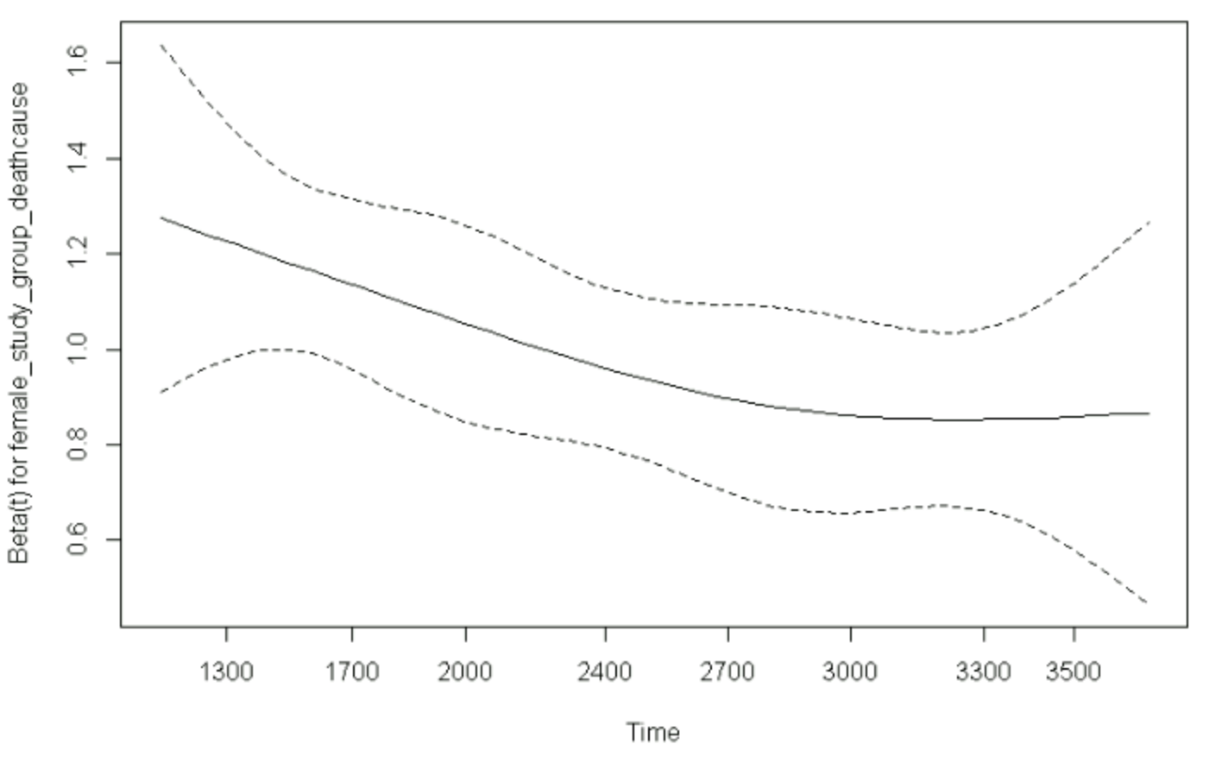


1. Log hazard ratio from the Cox model, which is estimated for males for years 3 to 10 of follow-up


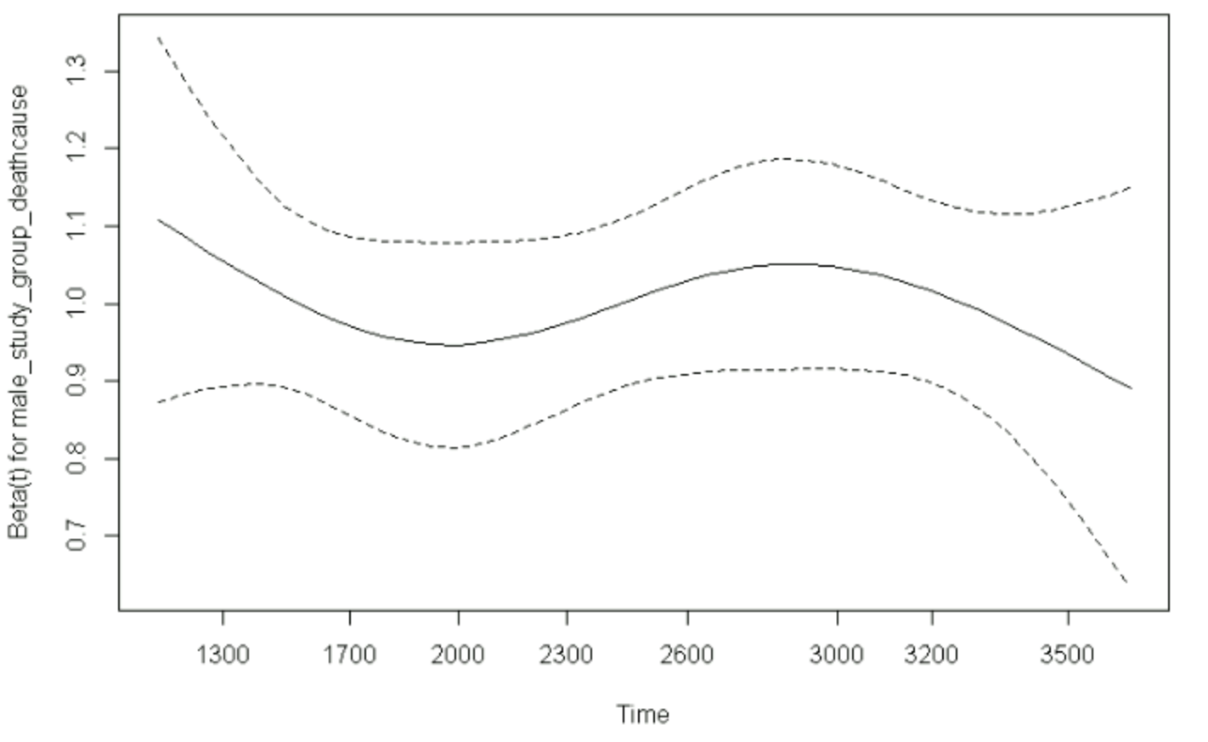


1. Log hazard ratio from the Cox model, which is estimated for females for years 10 to 20 of follow-up


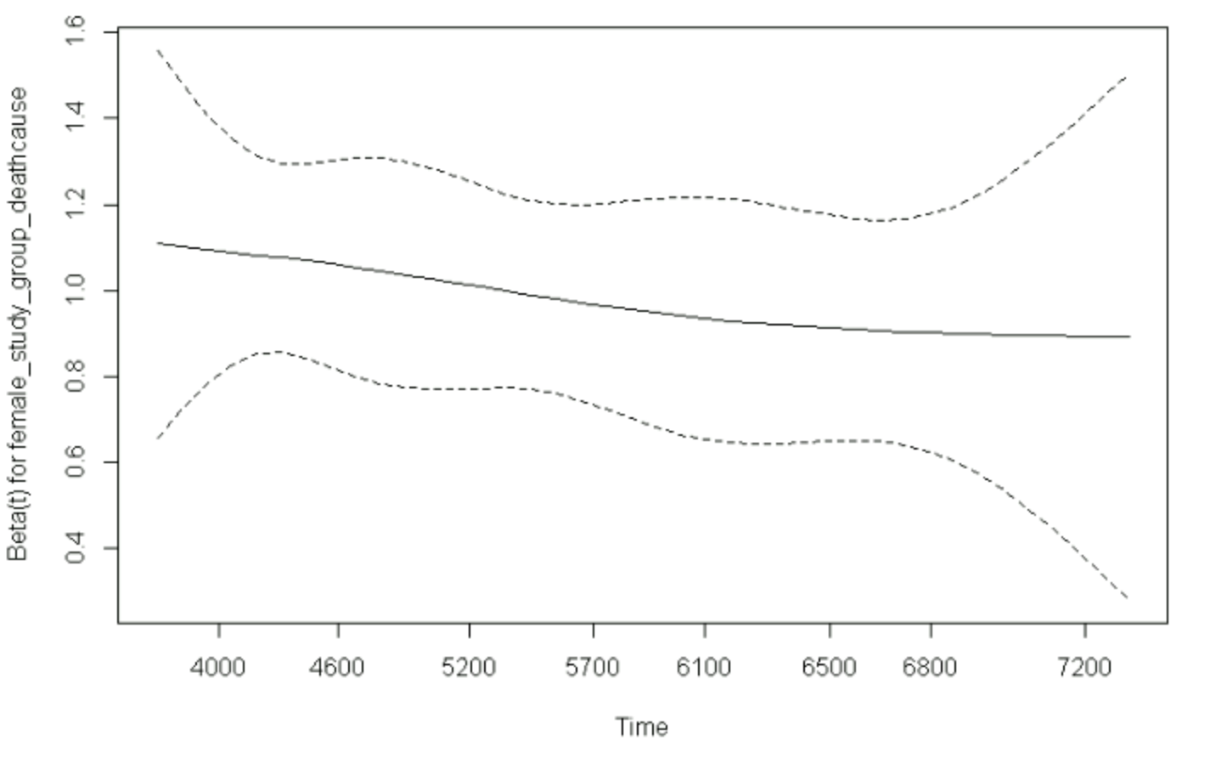


1. Log hazard ratio from the Cox model, which is estimated for males for years 10 to 20 of follow-up


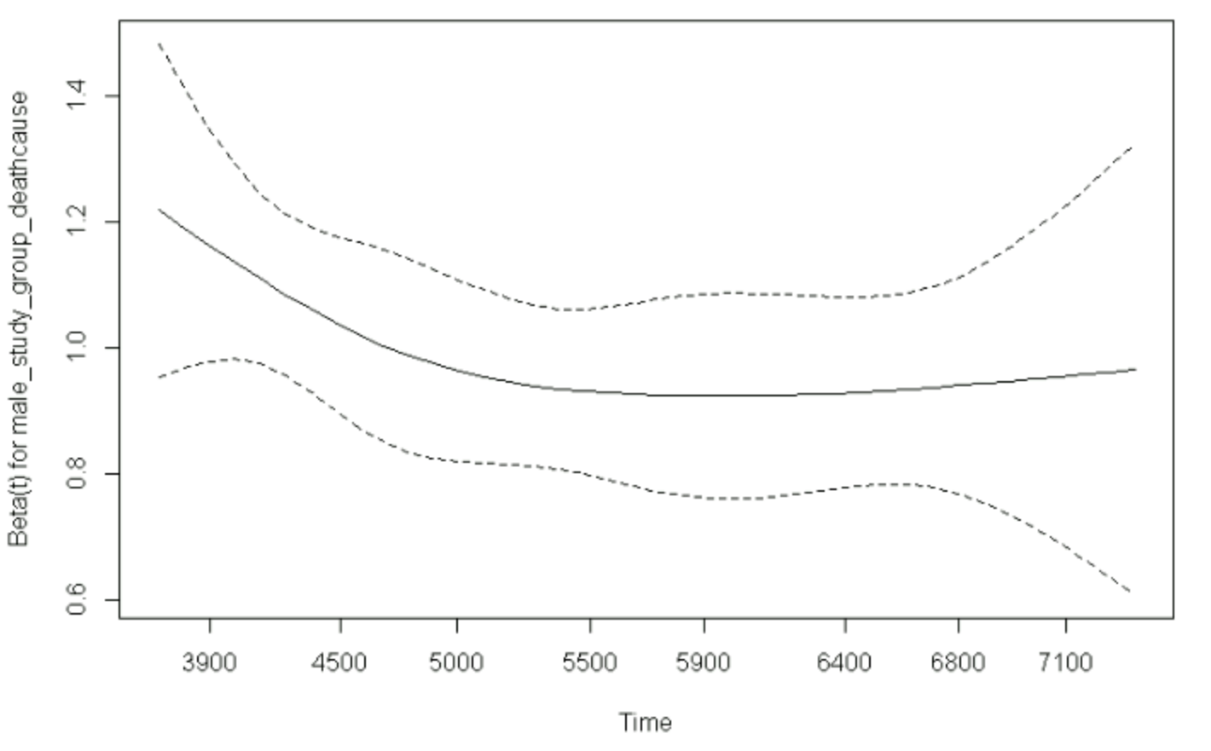


1. Log hazard ratio from the Cox model, which is estimated for married females for the follow-up period from 7 days to 20 years.


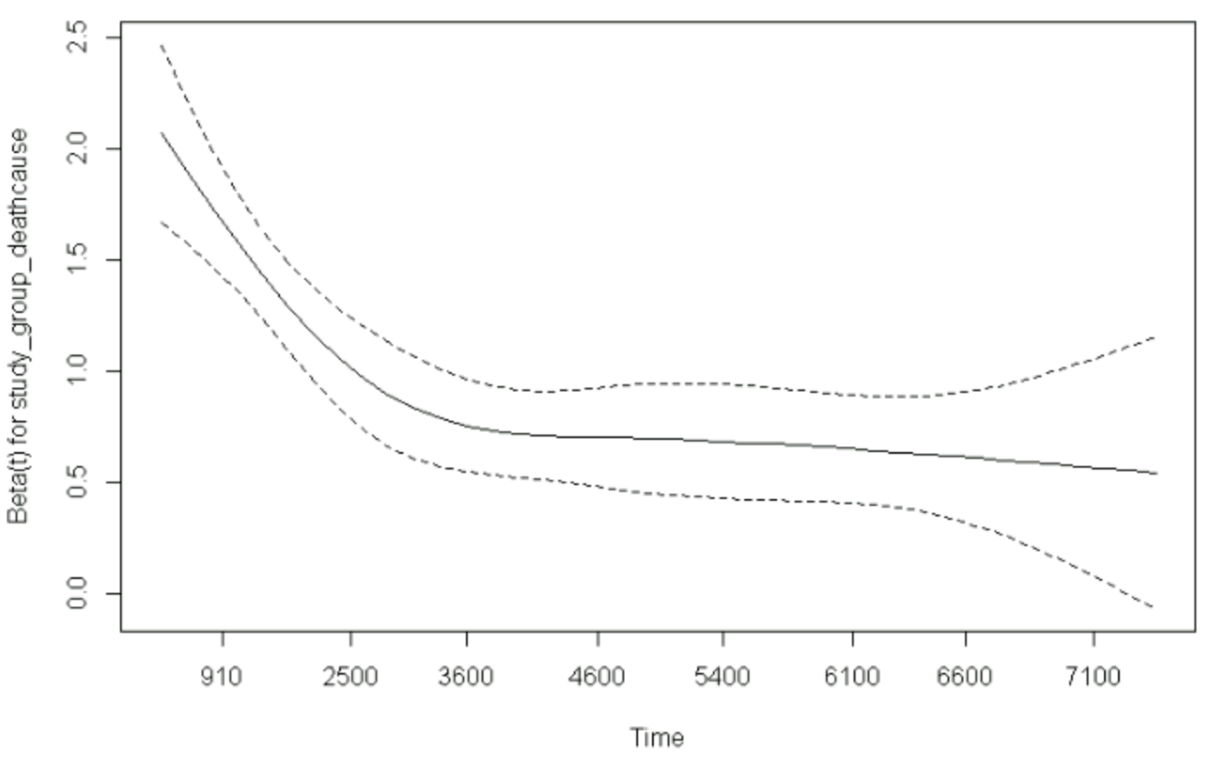


1. Log hazard ratio from the Cox model, which is estimated for married males for the follow-up period from 7 days to 20 years.


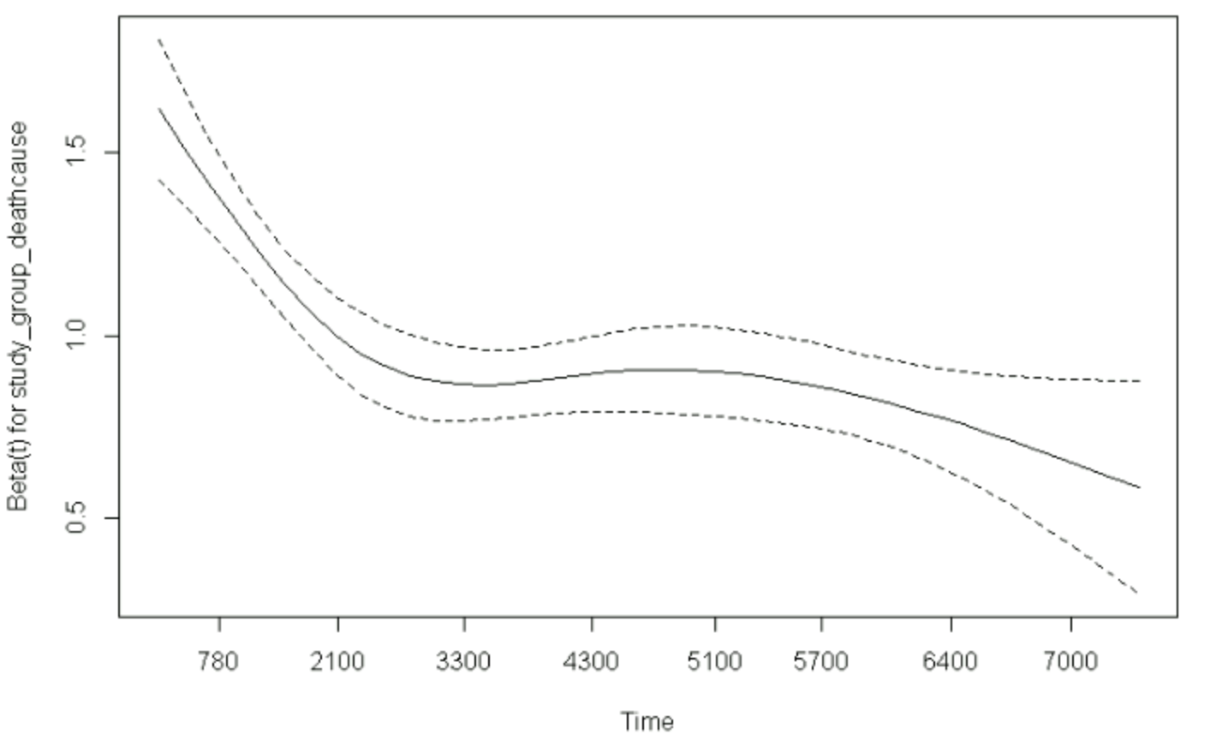


1. Log hazard ratio from the Cox model, which is estimated for cohabiting females for the follow-up period from 7 days to 20 years.


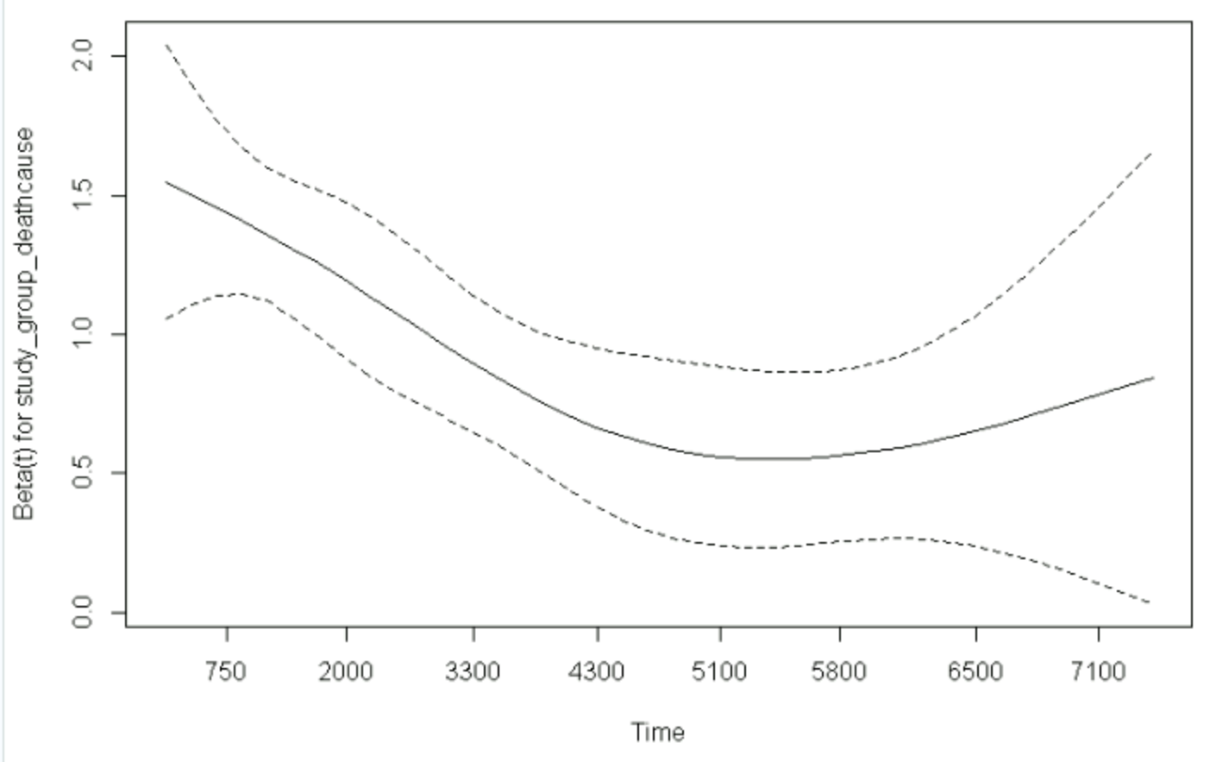


1. Log hazard ratio from the Cox model, which is estimated for cohabiting males for the follow-up period from 7 days to 20 years.


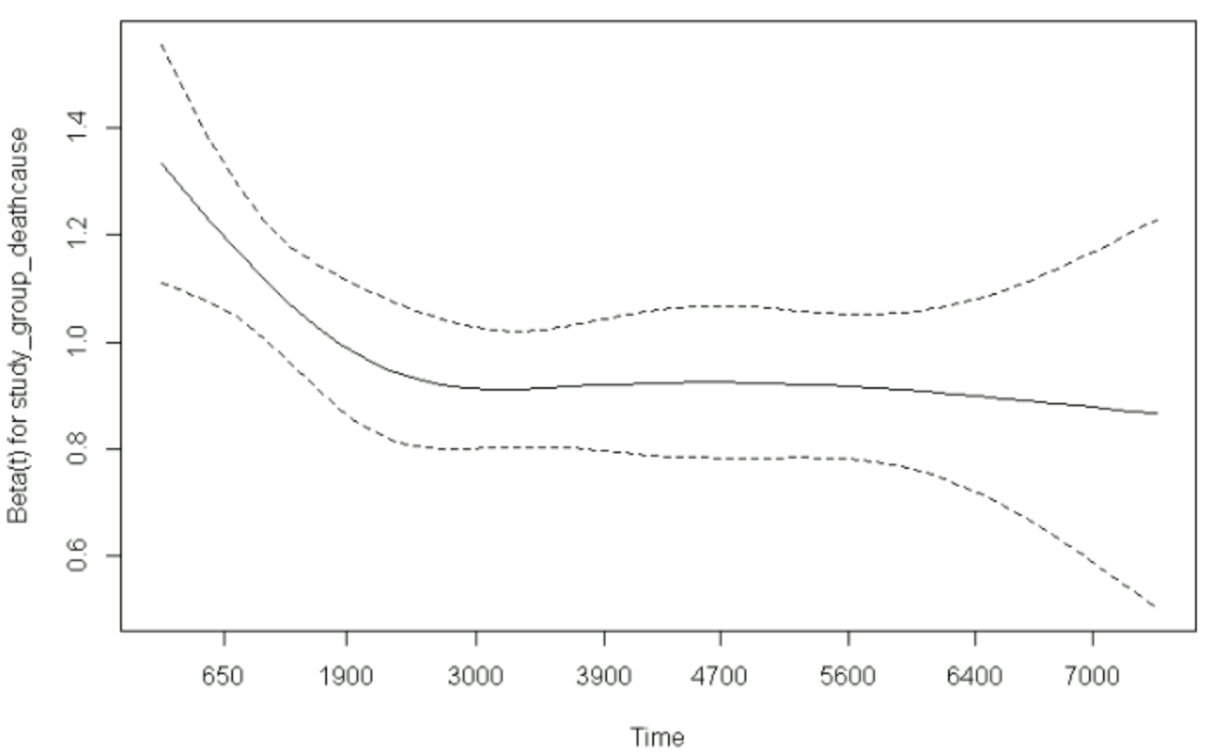


**Supplementary Figure 2.** Widowhood effect during 3-10 years after widowhood among married individuals and cohabitant partners, stratified by sex and cause of widowhood.


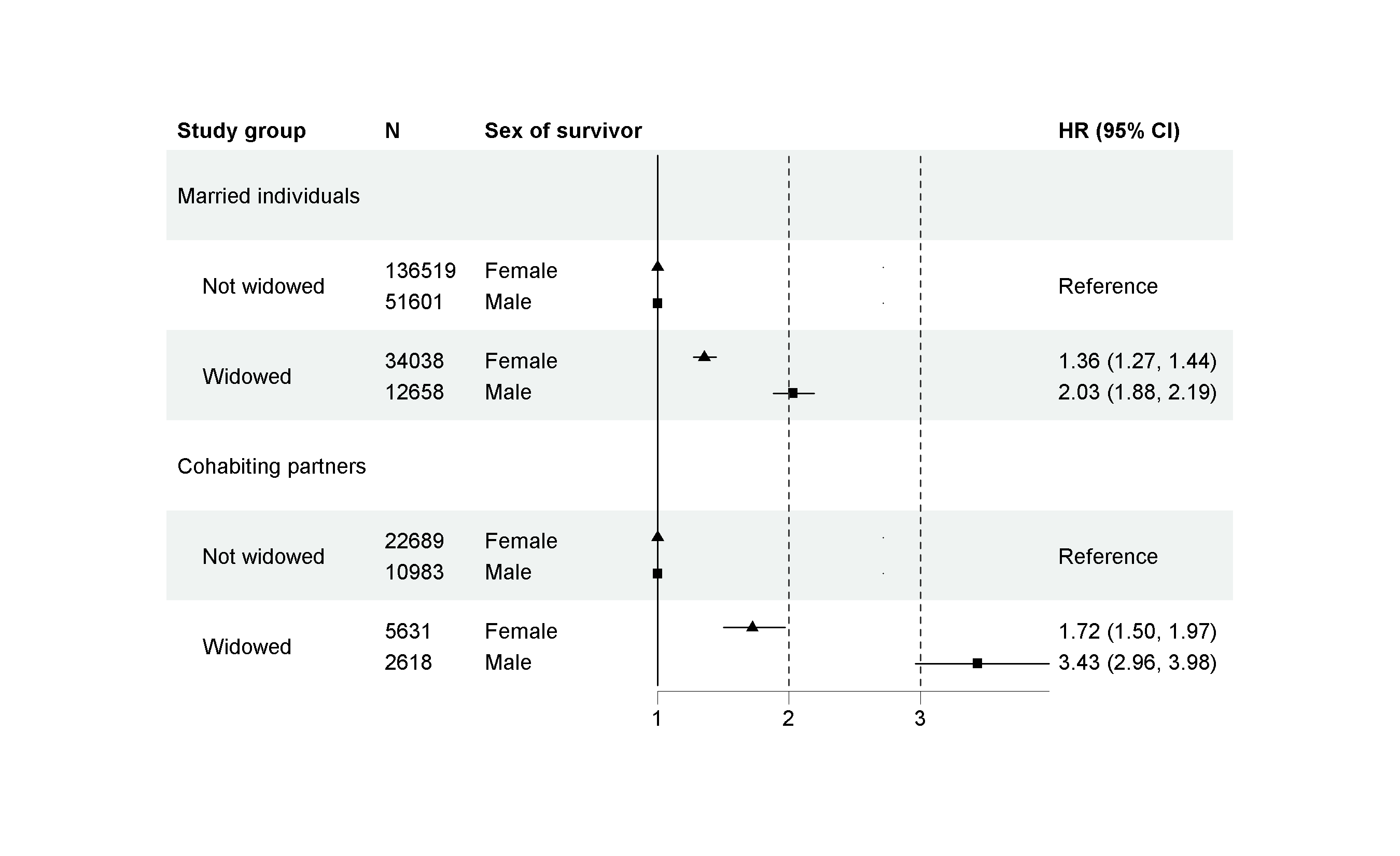


**Supplementary Figure 3.** Widowhood effect during 10-20 years after widowhood among married individuals and cohabitant partners, stratified by sex and cause of widowhood.


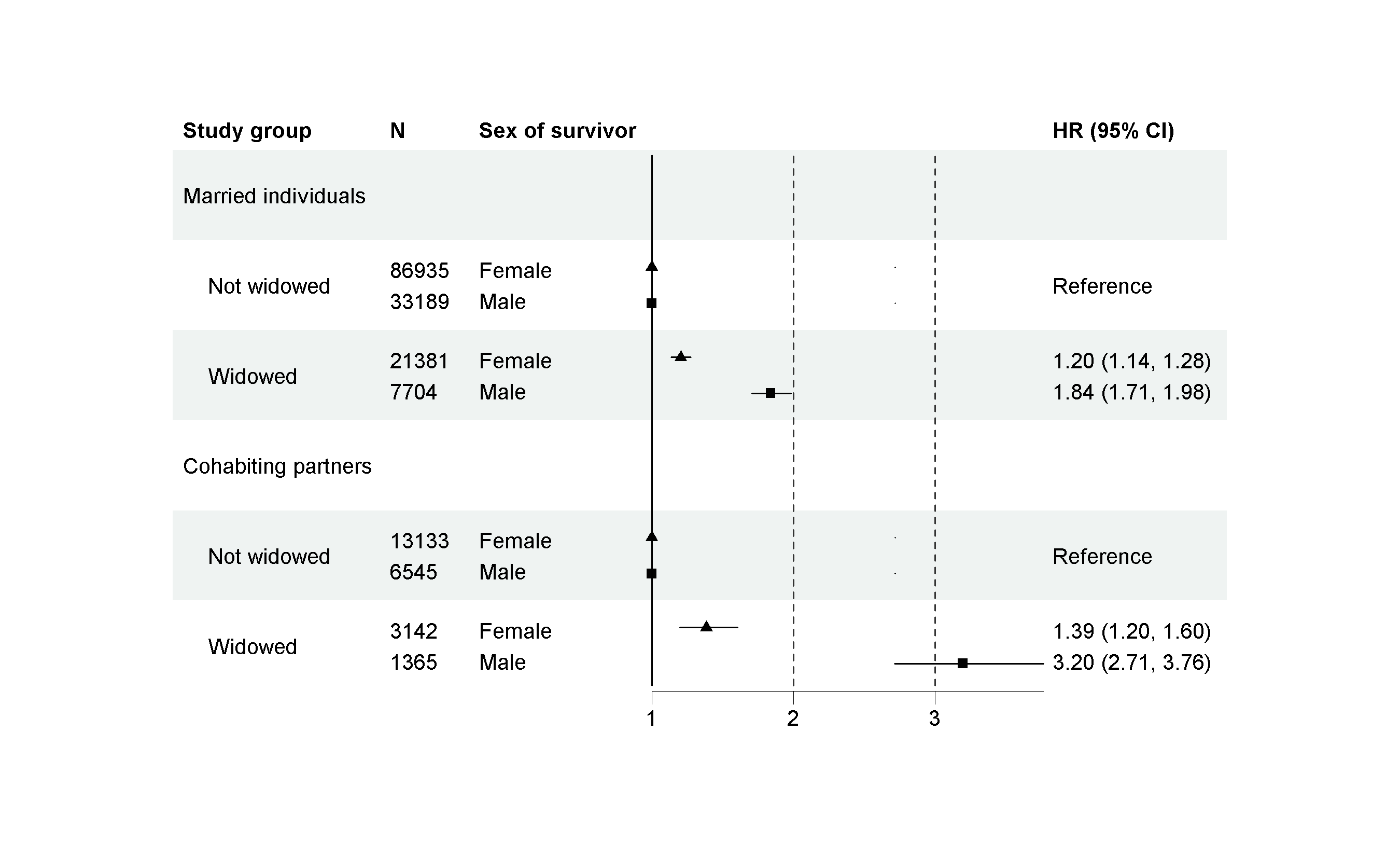


**Supplementary Figure 4.** Widowhood effect during the first three years after widowhood among married individuals and cohabitant partners, stratified by sex and the specific cause of widowhood.


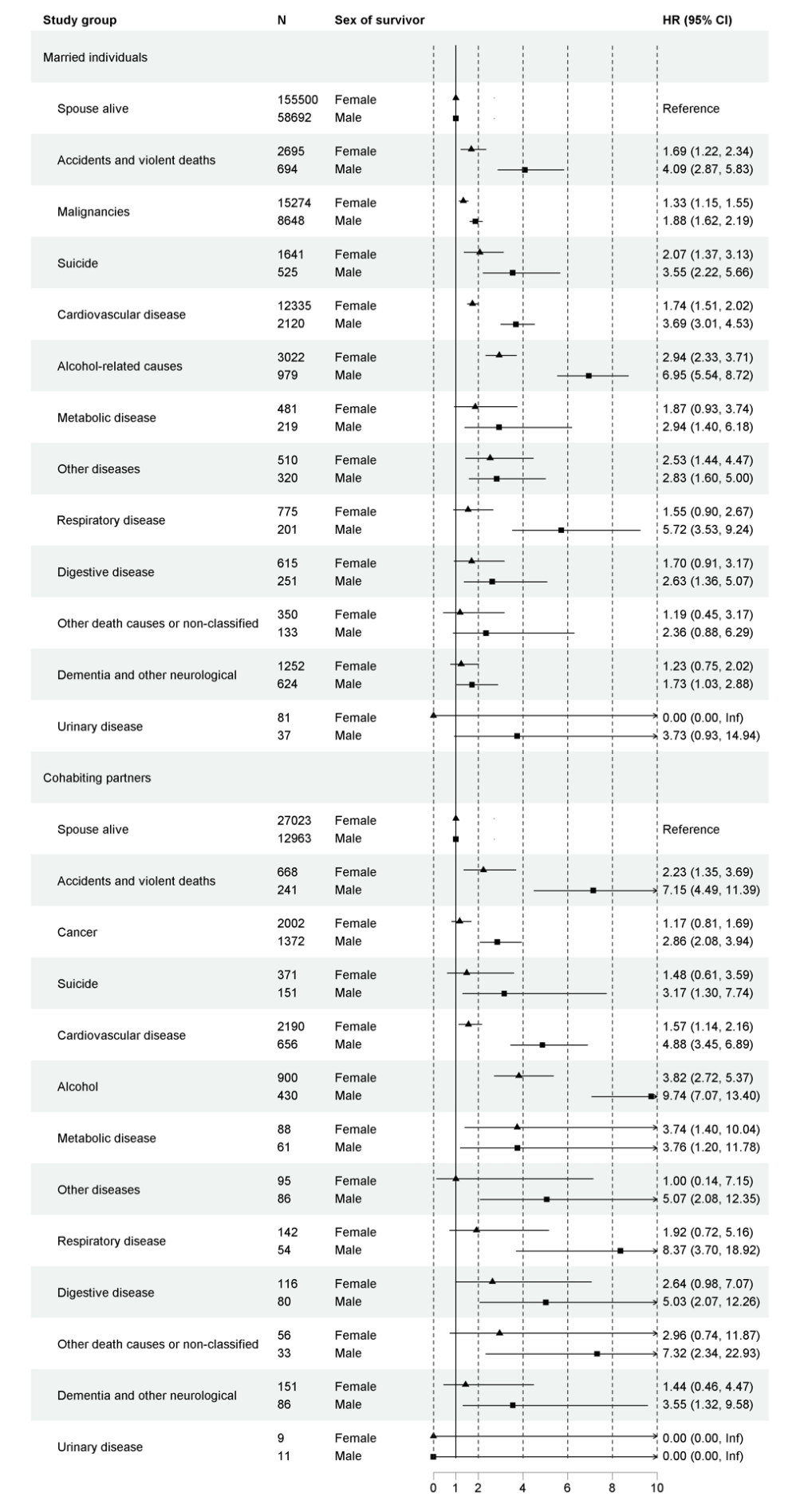


**Supplementary Figure 5.** Widowhood effect during 3-10 years after widowhood among married individuals and cohabitant partners, stratified by sex and cause of widowhood.


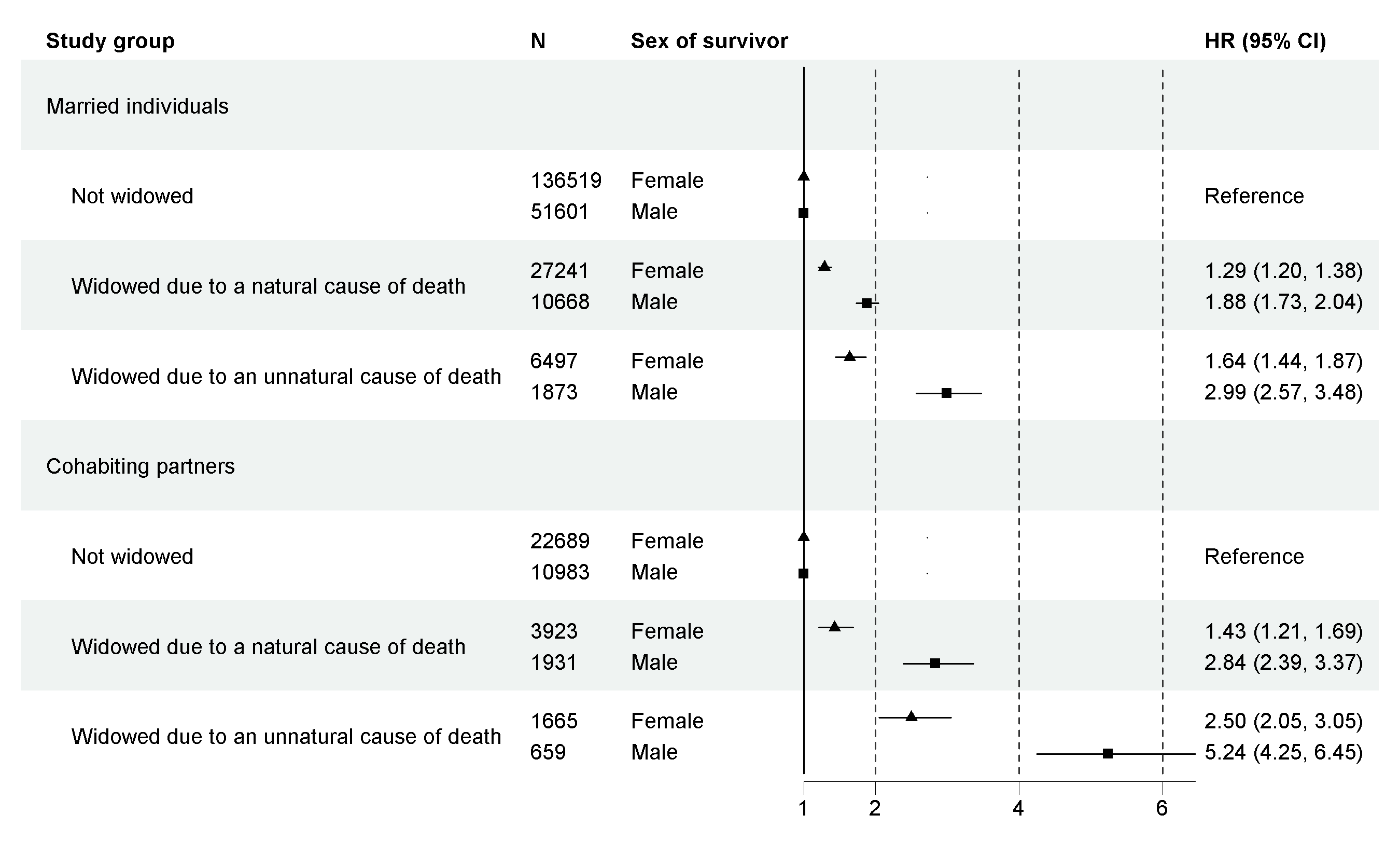


**Supplementary Figure 6.** Widowhood effect during 10-20 years after widowhood among married individuals and cohabitant partners, stratified by sex and cause of widowhood.


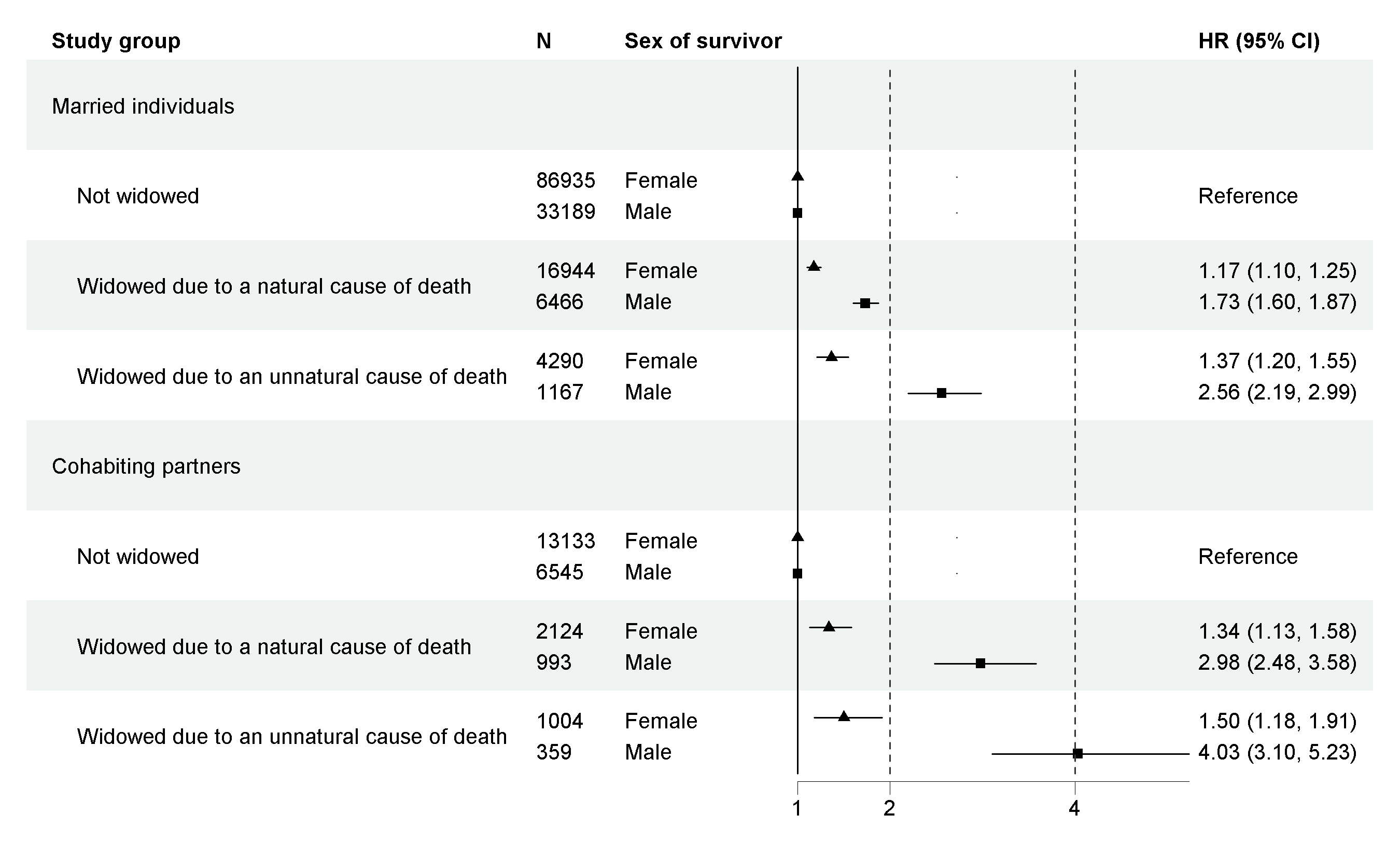


**Supplementary Figure 7.** Widowhood effect during 3-10 years after widowhood comparing widowed from a marriage and widowed from a cohabiting partnership to all controls, stratified by sex and cause of widowhood.


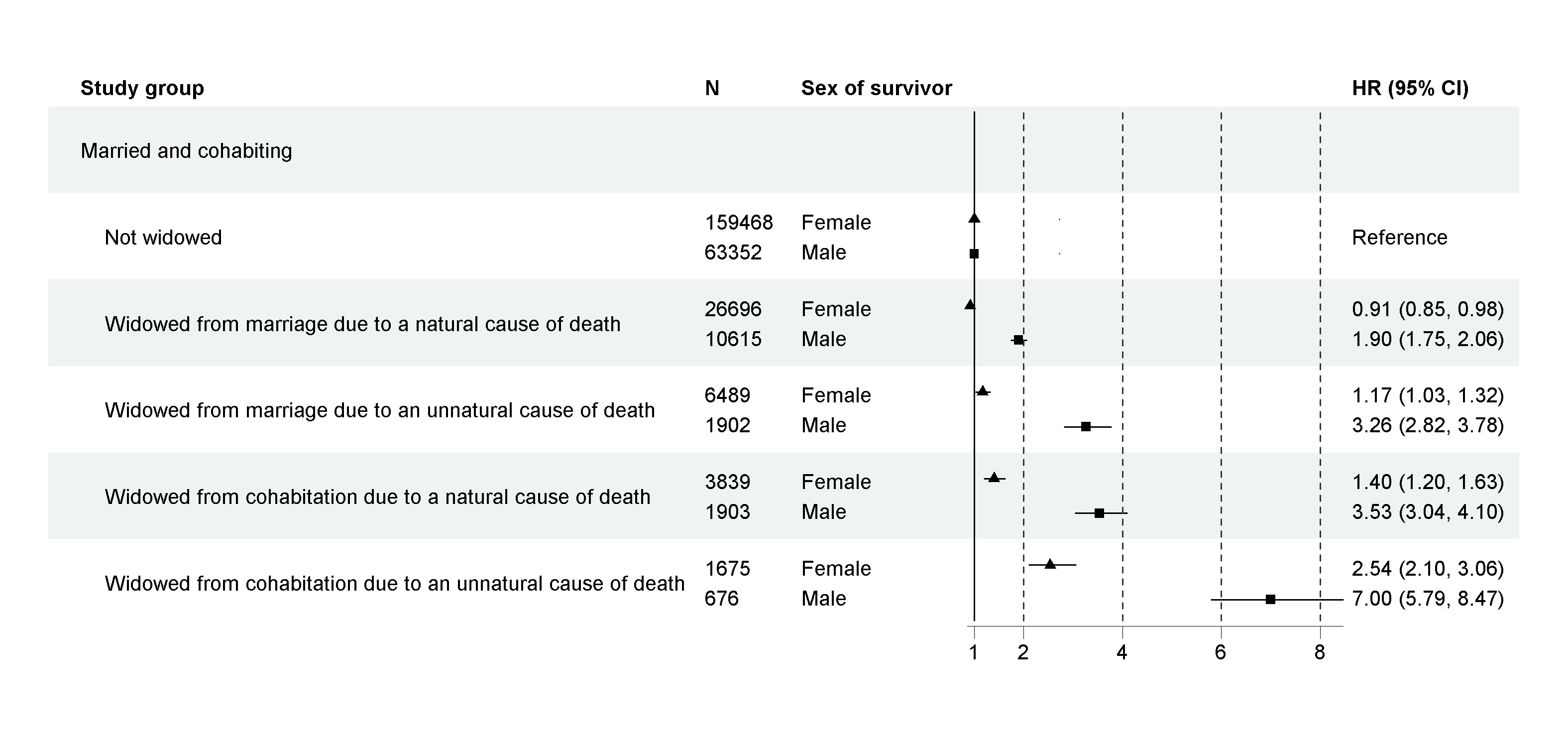


**Supplementary Figure 8.** Widowhood effect during 10-20 years after widowhood comparing widowed from a marriage and widowed from a cohabiting partnership to all controls, stratified by sex and cause of widowhood.


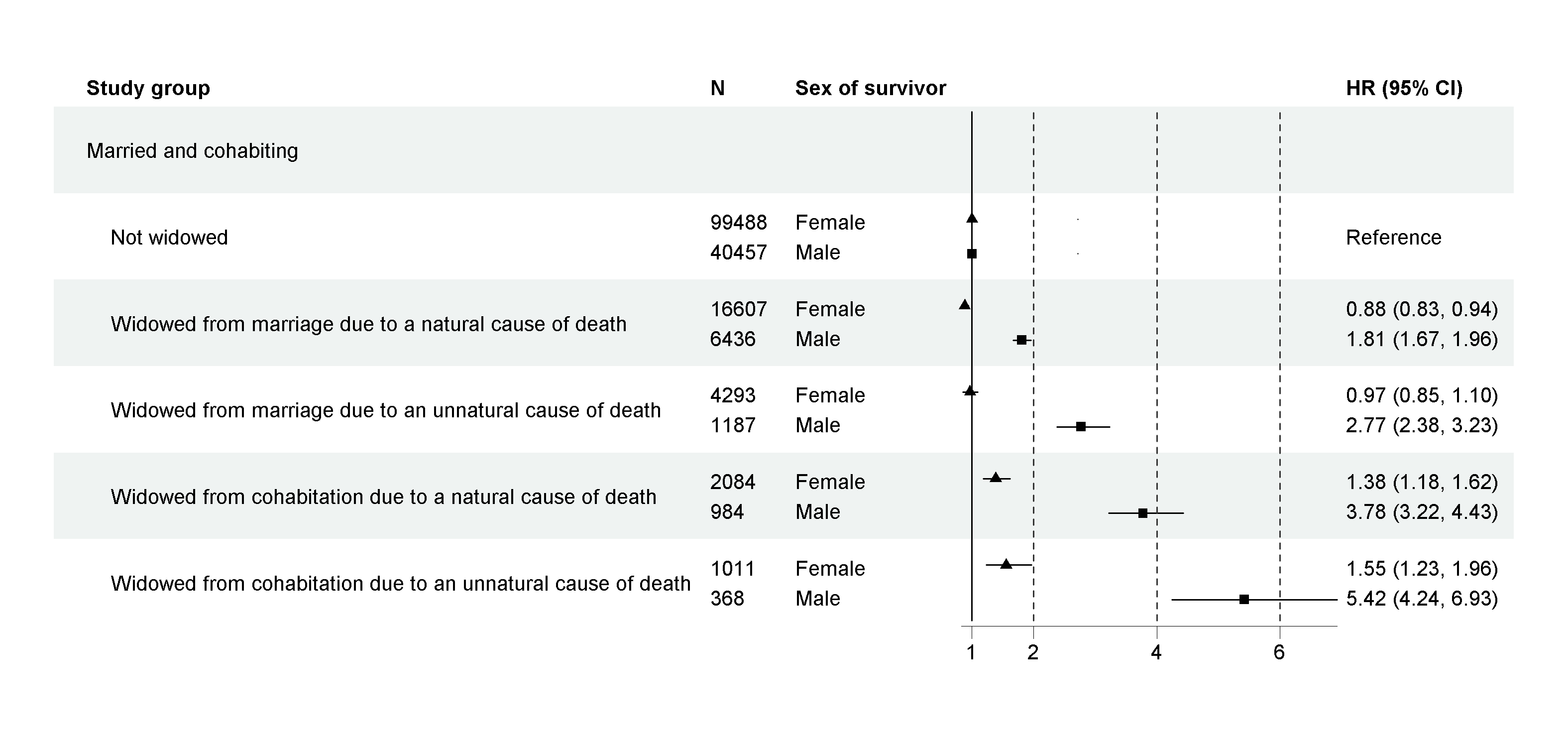


**Supplementary Figure 9.** Widowhood effect during 3-10 years after widowhood among married individuals and cohabitant partners, stratified by sex and re-partnering status after widowhood.


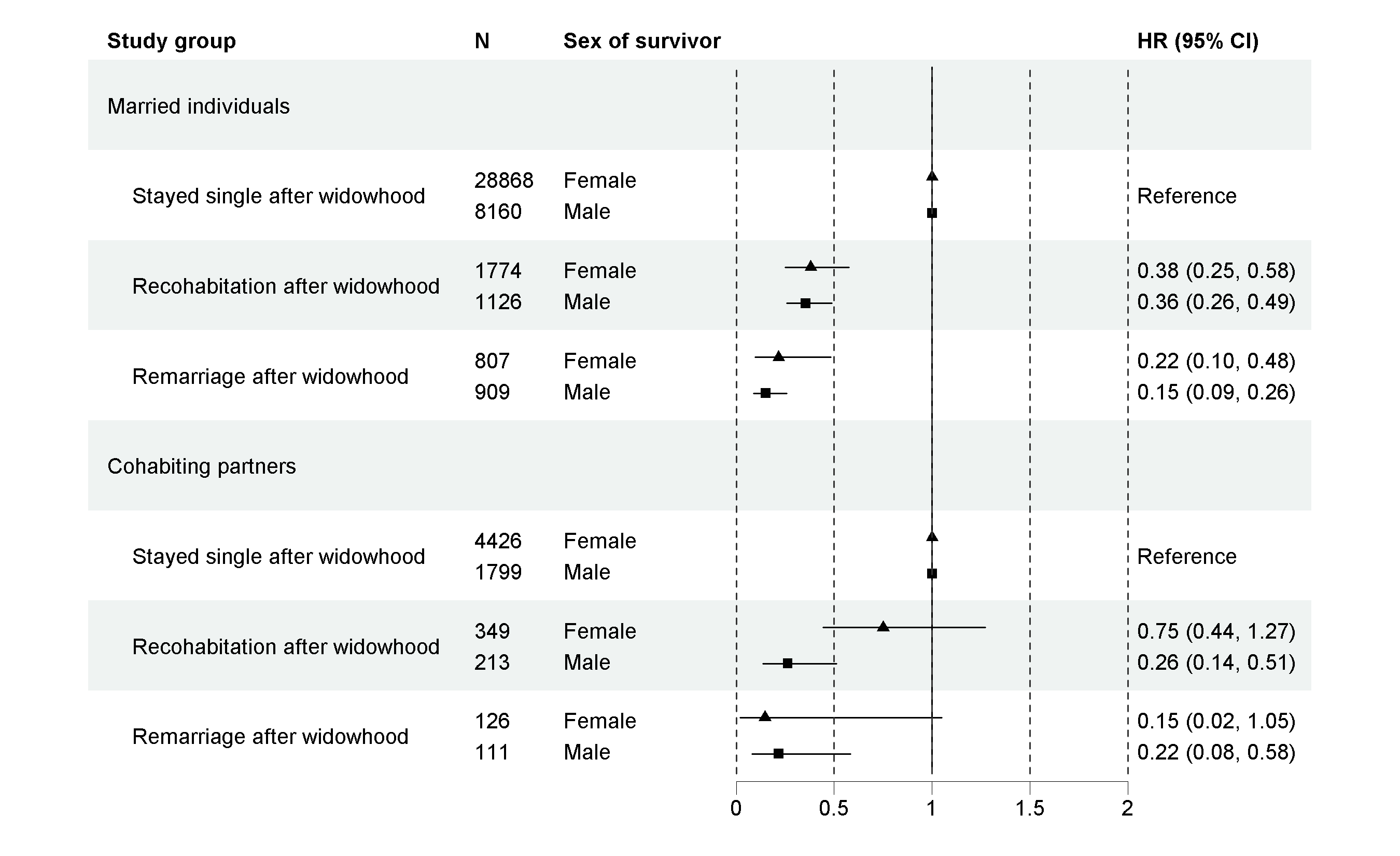


**Supplementary Figure 10.** Widowhood effect during 10-20 years after widowhood among married individuals and cohabitant partners, stratified by sex and re-partnering status after widowhood.


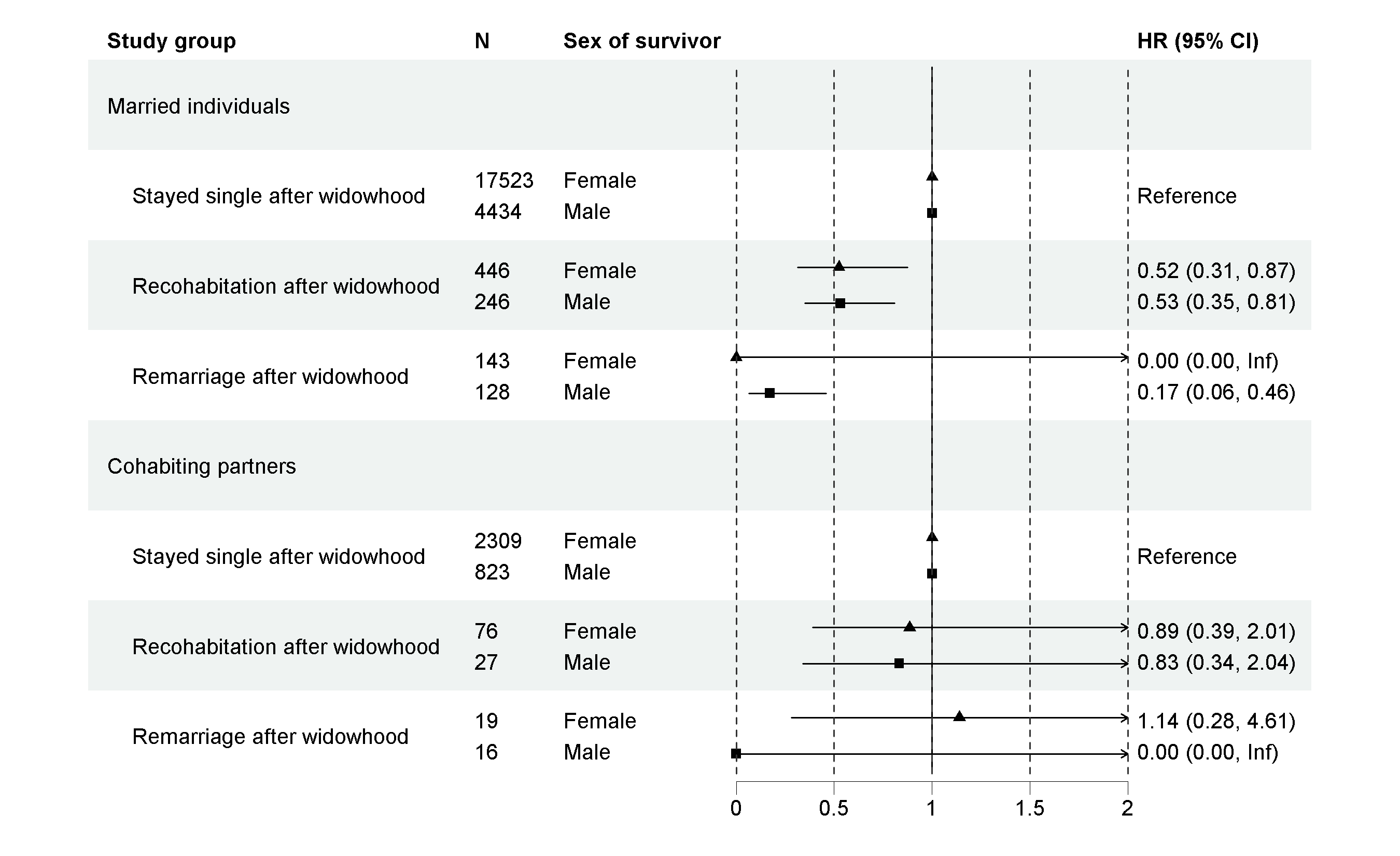

Supplement: gbaf164_Supplementary_Data [file gbaf164_supplementary_data.zip › JGSS suppl Korhonen, Leho, & Rissanen.docx]
